# Patterns of Objectively Measured Physical Activity in Normal Weight, Overweight, and Obese Individuals (20–85 Years): A Cross-Sectional Study

**DOI:** 10.1371/journal.pone.0053044

**Published:** 2013-01-07

**Authors:** Bjørge Herman Hansen, Ingar Holme, Sigmund Alfred Anderssen, Elin Kolle

**Affiliations:** Department of Sports Medicine, Norwegian School of Sport Sciences, Oslo, Norway; Fundación para la Prevención y el Control de las Enfermedades Crónicas No Transmisibles en América Latina (FunPRECAL), Argentina

## Abstract

**Background:**

The magnitude of the association between physical activity (PA) and obesity has been difficult to establish using questionnaires. The aim of the study was to evaluate patterns of PA across BMI-defined weight categories and to examine the independent contribution of PA on weight status, using accelerometers.

**Methods:**

The study was a cross-sectional population-based study of 3,867 adults and older people aged 20–85 years, living in Norway. PA was assessed for seven consecutive days using the ActiGraph GT1M accelerometer. Anthropometrical data was self-reported and overweight and obesity was defined as having a body mass index (BMI) of 25–<30 and ≥30 kg/m^2^, respectively.

**Results:**

Overweight and obese participants performed less overall PA and PA of at least moderate intensity and took fewer steps, compared to normal weight participants. Although overall PA did not differ between weekdays and weekends, an interaction between BMI category and type of day was present, indicating a larger difference in overall PA between BMI categories on weekends compared to weekdays. Obese participants displayed 19% and 25% lower overall physical activity compared to normal weight participants, on weekdays and weekends, respectively. Participants in the most active quintile of overall PA had a 53% lower risk (OR 0.47, 95% CI: 0.37 to 0.60) for having a BMI above or below 25 kg/m^2^, and a 71% lower risk (OR: 0.29, 95% CI: 0.20 to 0.44) for having a BMI above or below 30 kg/m^2^.

**Conclusions:**

Overweight and obese participants engaged in less overall PA and moderate and vigorous PA compared with normal weight individuals. The weight related differences in overall PA were most pronounced on the weekend and the risk of being overweight or obese decreases across quintiles of PA.

## Introduction

The adverse effects of overweight and obesity on health are well documented [1]. The prevalence of overweight and obesity has reached epidemic proportions worldwide [2], and Norwegian data indicate that 44% of women and 65% of men (aged 40–42 years) are either overweight or obese [3]. Although obesity is a complex disorder, a secular decrease in energy expenditure is believed to be an important contributor to both the development and maintenance of obesity [4], [5].

Nutrition surveys conducted in Norway in the past decades show that energy intake has not increased substantially [6], whereas average weight and prevalence of overweight and obesity have increased, during the same time period [3]. Although the composition of available foods may have changed, the increase in weight can be explained at least partly by a gradual decrease in habitual physical activity (PA), most notably by the apparent transition in occupational PA demands [7] and by increased car use and time spent at screen-based entertainment [8], [9].

Although the extent to which PA affects body composition has been evaluated comprehensively and there is generally an inverse relationship between PA and body weight [8], [10]–[16], the true magnitude of the association might be attenuated by a lack of precision in the measurement of PA and body composition [17]–[20]. Objective assessment of PA using activity monitors such as accelerometers can overcome many of the challenges related to self-reported measures of PA because they are unobtrusive and capable of accurately documenting the degree, nature, and pattern of PA [21], [22].

Accelerometers have been applied in large population-based studies of adults and older people and showed that overall PA, intensity-specific PA and time spent being sedentary differed according to body mass index (BMI) [23]–[26]. However, no studies of objectively assessed PA in a nationally representative Norwegian sample of adults and older people exist. The study will extent current knowledge by including analyses regarding differences in activity patterns between BMI-categories and the individual contribution of PA on the risk of being overweight or obese. Detailed information on the differences across BMI-categories in the amount of overall PA, intensity-specific PA, sedentary behaviour, as well as the patterns of PA is vital for developing our understanding of the aetiology of obesity, and will be useful for planning interventions to prevent weight gain and to increase PA in the general population.

The aim of the present study was to examine the relationship between PA and BMI by; 1) describing overall PA and intensity-specific PA across BMI categories; 2) evaluating the hourly patterns of overall PA stratified by BMI category across weekdays and weekend days 3) determining the independent contribution of overall PA and MVPA on weight status.

## Methods

### Ethics Statement

All participants provided written informed consent and the study was approved by the Regional Committee for Medical Ethics and the Norwegian Social Science Data Services AS.

### Study Design and Sample

The study was a cross-sectional multicentre study involving 10 test centres throughout Norway. Representative samples of 11,515 invitees (20–85 years) from the areas surrounding each test centre were randomly sampled from the Norwegian population registry. The study information and informed consent form were distributed via mail to the representative sample; 267 invitations were returned because of an unknown address, resulting in an eligible sample of 11,248 individuals. Written informed consent was obtained from a total of 3,867 individuals (34%). A total of 382 did not return any data. Because this study focused on BMI-defined weight categories, we excluded six women who self-reported pregnancy, giving a final sample of 3,479 (53% women) individuals. Of the final sample, 86 individuals did not wear the accelerometer, 14 had a defective monitor, 118 were excluded for providing fewer than 4 days of valid accelerometer data, and 171 reported no height and/or weight. A total of 3,090 (89% of the final sample) individuals were included in the association analysis.

### Assessment of PA

We used the ActiGraph GT1M accelerometer (ActiGraph, LLC, Pensacola, FL, USA) to assess each participant’s PA level. This micro-electro-mechanical system accelerometer is lightweight (27 g) and small (3.8 cm×3.7 cm×1.8 cm) and comprises a solid state monolithic accelerometer that uses microprocessor digital filtering. The accelerometer registers vertical acceleration as the number of counts per user-defined sampling interval (epoch), providing the researcher with a measure of overall PA (mean counts per minute; CPM) and intensity specific PA (number of time units with a mean count per time unit below or above a given threshold). Steps taken per day (steps/day) are also reported as a function of the “threshold crossing mode” embedded in the accelerometer, which counts the number of times the acceleration-generated signal crosses through the baseline reference each epoch and, according to the manufacturer, is representative of the number of steps taken.

Each participant received pre-programmed accelerometer and questionnaire by mail. Standardized instructions included information about wearing the accelerometer in an adjustable cotton fabric belt over the right hip for seven consecutive days, and removing it for water activities such as showering and swimming. After registration, the participants returned the accelerometer and questionnaire by mail to their respective test centre.

### Accelerometer Data Handling

Accelerometers were initialized and downloaded using software provided by the manufacturer (ActiLife, ActiGraph). Data were collected in 10-s epochs. The 10-s epochs were collapsed into 60-s epochs for comparison with other studies. The data were reduced to derivative variables with customized SAS-based macros (SAS Institute Inc., Cary, NC, USA), and included if the participant had accumulated at least 10 h of valid activity recordings per day for at least 4 days. Time periods of at least 60 consecutive minutes with zero counts, with allowance for 1 minute with counts above zero, was defined as non-wear time and thus, wear time was defined by subtracting non-wear time from 18 hours (all data between 00∶00 and 06∶00 were excluded to avoid the potential bias of participants wearing the monitor while sleeping). In addition to overall PA and steps/day, all time awake was categorised by intensity according to the specific activity CPM values. In particular, light intensity PA was defined by counts between 100 and 2,019 CPM, moderate intensity PA as counts between 2,020 and 5,999 CPM and 6,000 CPM represents the lower threshold for vigorous intensity activities [27]. Time spent at <100 CPM (not counting non-wear time) was classified as sedentary behaviour. Bouts of moderate-to-vigorous PA (MVPA) was calculated by summing all activity ≥2020 counts per minute that occurred in sustained bouts of at least 10 min (with allowance for one or two interruptions). To establish patterns of overall PA, minute-by-minute activity counts were summed for each hour of measurement for weekdays and weekend days, respectively.

### Other Measures

Height and weight were self-reported by questionnaire and BMI was computed as weight (kg) divided by meters squared (m^2^). BMI was categorized according to the guidelines set forward by the World Health Organization, with overweight and obesity defined as a BMI of 25–<30 and ≥30 kg/m^2^, respectively [2]. Because of the small sample size, underweight participants (n = 35) were included in the normal weight category; this did not cause any significant change in overall PA for the normal weight participants. To assess health status, participants were asked to rate their perceived health status as very good, good, either, poor, or very poor. Because of the low prevalence of poor health (n = 104, 3.0%) and very poor health (n = 3, 0.1%), the answers were grouped into two categories for the analysis; very good/good and either/poor/very poor. Educational attainment was categorized into four groups: less than high school, high school, less than 4 years of university, and university for 4 years or more. Smoking habits were reported and dichotomized before the variable was entered into the analysis (smoking vs. not smoking). In order to register the amount of certain activities poorly registered by the accelerometers, participants also answered a 1-page questionnaire assessing the amount of cycling, swimming and muscular strength training performed during the 7-day registration period.

### Statistical Analyses

The descriptive data are presented according to sex specific BMI categories as percentage, mean, and standard deviation (SD) or standard error of the mean (SE), and 95% confidence interval (CI) where appropriate. Student’s *t*-test for independent groups was used to identify differences in anthropometric data between sexes. Chi-square tests were used to test for differences in self-reported health and level of education between weight categories. One-way analyses of covariance adjusting for age and test centre, with the Bonferroni post hoc tests, were performed to identify within-sex differences in PA between BMI categories.

A one-way repeated measurement analysis was conducted to explore whether the impact of type of day (weekday or weekend) differed across BMI category (normal weight, overweight and obese). Type of day was defined as the repeated factor in the analysis, with weight category as the between-subject factor, and age, sex and test centre as covariates. A Wilks` Lambda with a significance level of p<0.05 indicated a significant interaction effect between BMI category and type of day.

Logistic regression was performed to assess the impact of a number of factors on the likelihood that participants were either overweight or obese (classified as having a BMI ≥25 kg/m^2^) or obese (BMI≥30 kg/m^2^). The independent variables included in the model were age, sex, level of education, self-reported health, smoking, and quintiles of either CPM or MVPA. These variables were included because of their known association to body weight. For the logistic regression, CPM and MVPA was categorized into quintiles and assigned ascending values where 1 was the least active group and 5 the most active group. A significant interaction was found between self-reported health and quintile of PA (*p = *0.016). However, stratifying by health status did not change the direction of the relationship or the magnitude substantially and for sake of simplicity, the variable was included in the model and treated as a potential confounder. A total of 4 regression analyses were performed (quintiles of CPM and risk of BMI ≥25 kg/m^2^, quintiles of CPM and risk of BMI ≥30 kg/m^2^, quintiles of MVPA and risk of BMI ≥25 kg/m^2^, and quintiles of MVPA and risk of BMI≥30 kg/m^2^). The resulting odds ratios are displayed graphically as reduction in relative odds (%). All statistical analyses were performed using PASW Statistics 18 for Windows (IBM Corporation, Route, Somers, NY, USA) and a two-tailed alpha level of 0.05 was used for statistical significance.

## Results

The physical characteristics of the participants with complete anthropometric data are presented in Table 1. The prevalence of overweight and obesity was 30% and 11% for women, and 47% and 13% for men. Health status differed according to weight status. Although 82% of normal weight individuals reported having at least good health, the corresponding percentages were 75% for overweight and 58% for obese individuals.

**Table 1:** Descriptive data for participants (SD) by weight category.

	Weight category											
	Normal weight				Overweight				Obesity			
	Women		Men		Women		Men		Women		Men	
n (%)	1046	(60)	638	(41)	519	(30)	707	(47)	190	(11)	206	(13)
Age (years)	47.5	(15.5)	49.6	(16.4)	50.5	(14.1)	51.0	(14.2)	48.5	(13.6)	49.0	(13.3)
Height (cm)	167.2	(5.8)	180.3	(6.6)	166.6	(5.8)	180.0	(6.1)	165.6	(7.5)	179.6	(7.4)
Weight (kg)	62.2	(6.5)	75.2	(7.6)	75.3	(6.4)	88.0	(7.4)	91.8	(12.4)	105.1	(11.4)
BMI (kg/m^2^)	22.2	(1.8)	23.1	(1.5)	27.1	(1.4)	27.1	(1.4)	33.5	(4.4)	32.5	(2.4)
General health (%)												
Very good/Good	82.0		81.5		74.1		75.0		59.5		55.6	
Either/Poor/Very poor	18.0		18.5		25.9		25.0		40.5		44.4	
Education (%)												
Less than high school	11.3		12.8		15.9		13.7		13.2		17.2	
High school	32.1		35.4		42.2		41.3		42.6		51.5	
University <4 years	28.2		21.6		20.2		22.8		20.5		19.1	
University ≥4 years	28.3		30.2		21.7		22.3		23.7		12.3	

The number of valid days of activity recordings (6.8 days, data not shown) and daily wearing time (880 min, data not shown) did not differ between the weight categories. The measures of PA stratified by BMI category are presented in Table 2. Normal weight women had a higher overall PA level and steps/day compared with both overweight and obese women. The mean difference between normal weight and obese women was 76 CPM (95% CI: 51, 101) and 1,971 steps/day (95% CI: 1,412, 2,529). Overall PA and steps per day displayed a similar pattern for men, although only reaching statistical significance for overall PA. The mean difference in overall PA between normal weight and obese men was 78 CPM (95% CI: 50, 106).

**Table 2:** Measures of PA and sedentary behaviour (95% Confidence Intervals) stratified by BMI category.

	Weight category											
	Normal weight				Overweight				Obesity			
	Women		Men		Women		Men		Women		Men	
Overall PA (CPM)	352	(344, 360)	368	(357, 379)	324	(313, 336)**	331	(320, 314)**	276	(257, 295)**	290	(270, 310)**
Steps per day	8554	(8374, 8734)	9196	(8177, 10.214)	7789	(7532, 8046)**	8621	(7654, 9587)	6583	(6163, 7003)**	6980	(5179, 8780)
Sedentary behaviour (min)	528	(524, 533)	552	(546, 558)	529	(523, 534)	558	(552, 564)	546	(535, 557)*	574	(5.64, 585)**
Light PA (min)	304	(300, 309)	284	(278, 289)	310	(304, 317)	284	(278, 289)	301	(291, 312)	273	(263, 283)
Moderate PA (min)	33.3	(32.0, 34.6)	35.6	(33.9, 37.3)	28.4	(26.6, 30.2)**	32.2	(30.5, 33.8)*	21.7	(18.8, 24.7)*	27.0	(23.9, 30.0)**
Vigorous PA (min)	2.6	(2.3, 2.9)	4.0	(3.5, 4.6)	1.4	(1.1, 1.9)**	1.6	(1.1, 2.1)*	0.7	(0.0, 1.4)**	1.1	(0.2, 2.1)**
Bouts of MVPA (min)	21.0	(19.9, 22.2)	19.3	(17.7, 20.8)	15.7	(14.1, 17.3)**	15.4	(14.0, 16.9)**	10.4	(7.8, 13.1)**	13.2	(10.4, 15.9)**

All values are adjusted for test centre and age, and indicators of intensity-specific PA were additionally adjusted for mean daily wear time.

* *p*<0.05, compared with normal weight, within sex.

** *P*≤0.001, compared with normal weight, within sex.

Normal weight women and men spent an average of 8.8 and 9.2 h per day, respectively, being sedentary. The amount of time spent being did not differ between normal weight and overweight participants, but obese women and men spent an average of 17 min (95% CI: 3, 32) and 22 min (95% CI: 7, 37) more, respectively, pursuing sedentary behaviours. The amount of light PA did not differ between BMI categories, but PA of at least moderate intensity decreased significantly with increasing BMI.

**Figure 1:**
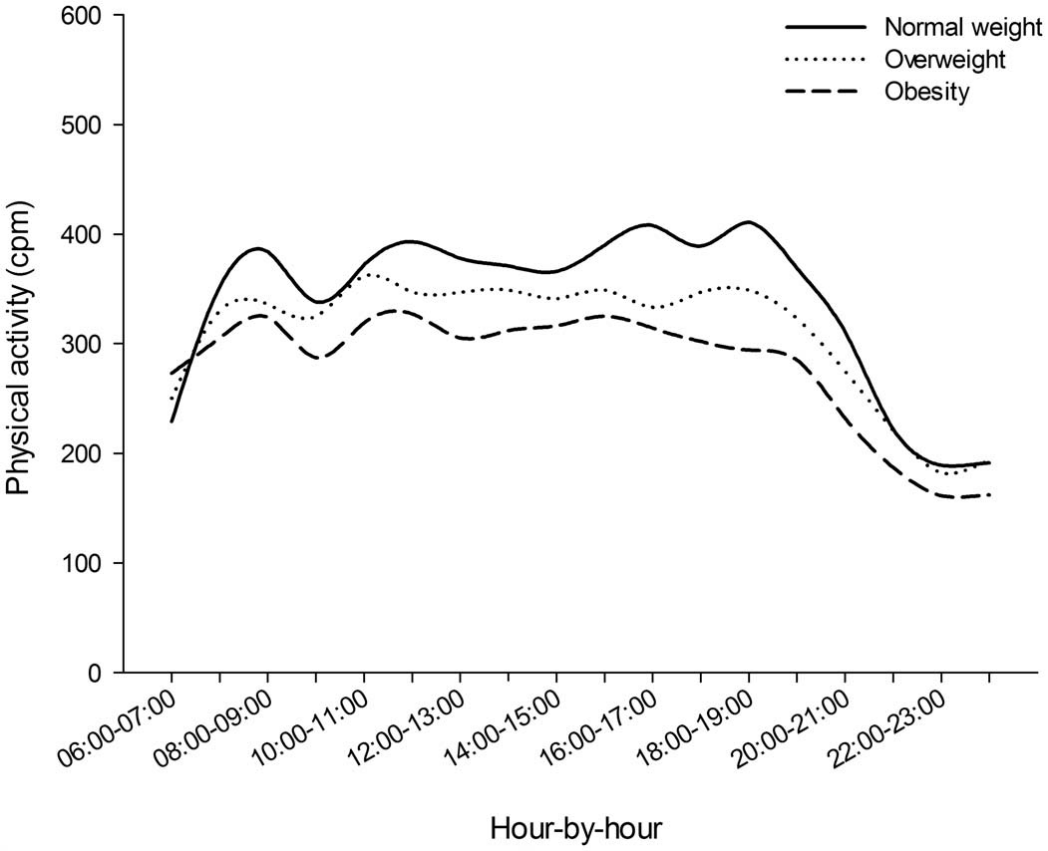
Hourly distribution of overall PA level (CPM) for normal weight, overweight and obese individuals on weekdays.

**Figure 2:**
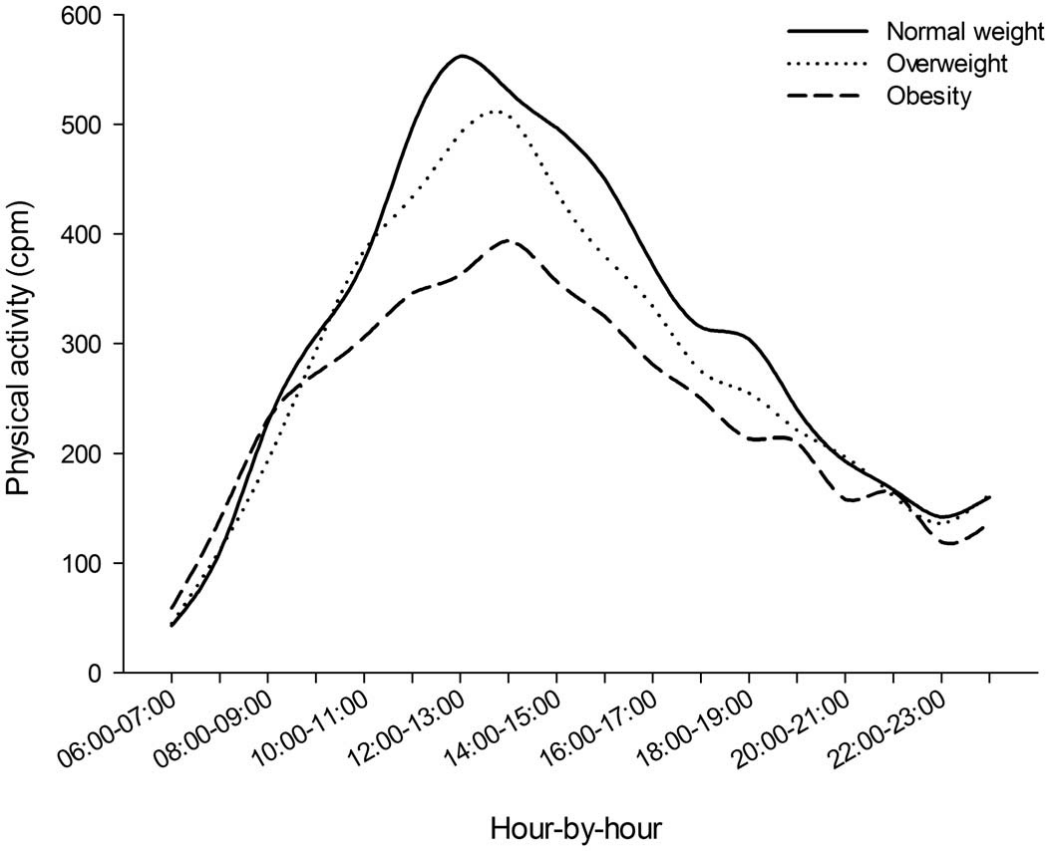
Hourly distribution of overall PA level (CPM) for normal weight, overweight and obese individuals on weekend days.

Overall PA decreased across BMI categories at both weekdays and weekends. However, a significant interaction (Wilks` Lambda 0.998, p = 0.042) was observed between type of day and weight category, indicating that the impact of type of day on overall PA differed between the BMI categories. Overall, differences in PA were larger between the BMI categories on weekends compared to weekdays. Compared to normal weight participants, obese participants displayed a 19.2% (355 CPM vs. 287 CPM) lower overall PA on weekdays, while similar difference on weekends 24.6% (370 CPM vs. 279 CPM). As displayed in Figure 1–2, these differences were particularly visible at around midday and early afternoon.

**Figure 3:**
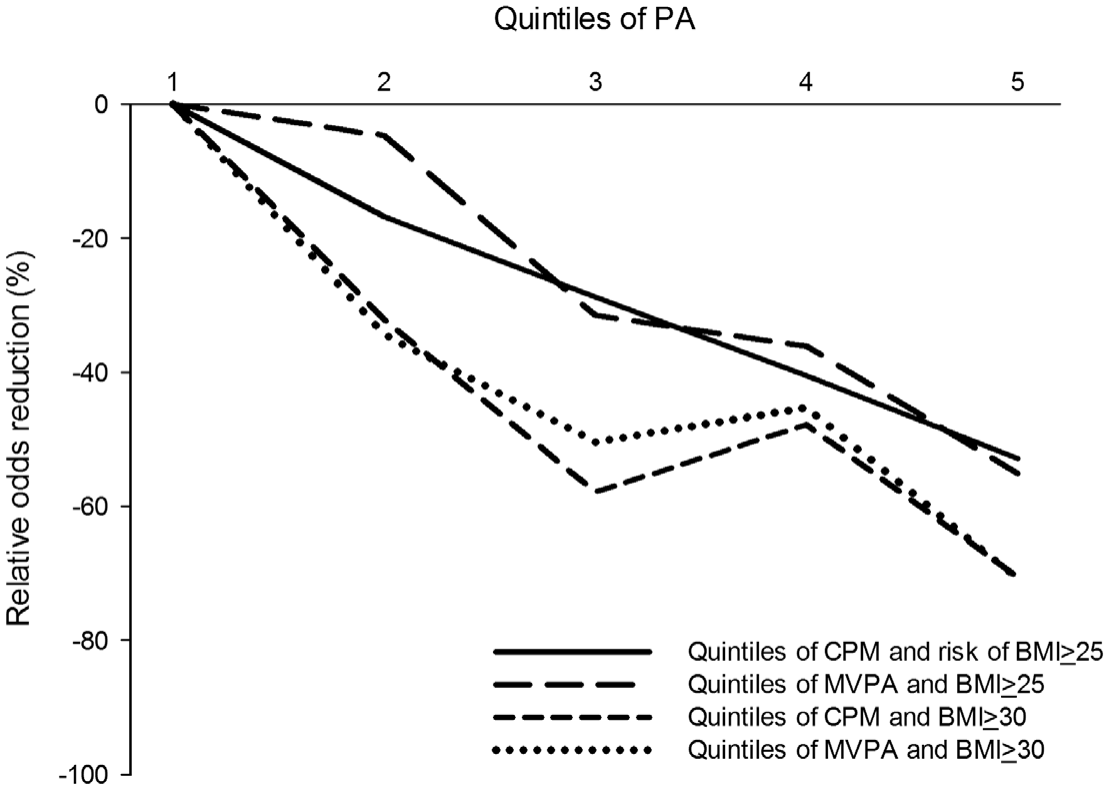
The reduction in relative odds for being overweight or obese associated with increased overall PA and MVPA (the models are adjusted for age, sex, level of education, smoking and self-reported health).

Logistic regression was performed to assess the impact of a number of factors on the likelihood that individuals would be either overweight or obese (Figure 3). The models containing all predictors were significant (*p*<0.001), indicating the ability to distinguish between normal weight, overweight and obese individuals. The model including quintiles of CPM explained between 8% (Cox and Snell R-squared) and 11% (Nagelkerke R-squared) of the variance in weight status. The models showed an increased odds ratio (OR) for being overweight or obese between quintiles of PA and the dose-response relationship was about linear (Figure 3). Participants in the most active quintile of overall PA had a 53% lower risk (OR: 0.47, 95% CI: 0.37 to 0.60) for having a BMI of 25 kg/m^2^ or above, and a 71% lower risk (OR: 0.29, 95% CI: 0.20 to 0.44) for having a BMI of 30 kg/m^2^ or above. Similar findings were observed for quintiles of MVPA.

## Discussion

The present study shows a consistent decrease in PA level with increasing BMI. Overweight and obese participants had a lower overall PA level, took fewer steps each day, and performed less daily moderate and vigorous PA and MVPA performed in bouts of ≥10 minutes than did normal weight participants. Obese participants also accumulated more sedentary time, compared with normal weight participants.

The results of the present study are consistent with those of studies that used accelerometers to measure PA in large populations of adults and older people. Tudor-Locke et al. (2010) showed that, among Americans, overall PA decreased consistently with increasing BMI and that men had a higher overall PA than women, within each BMI category. The gradient between BMI categories was similar in the present study, indicating that the decrease in overall PA with increasing BMI is a consistent finding. However, only negligible sex differences within each BMI category were observed in our study. Norwegian women are consistently more active than American women, whereas Norwegian men are consistently less active than American males across all BMI categories, independent of age [25]. This finding also agrees with Swedish data showing a similar decrease in overall PA with increasing BMI but no apparent sex difference within each BMI category [26].

The relative differences in PA between BMI categories in the present study were larger for intensity-specific PA than for the indicators of overall PA. Normal weight women performed twice as much MVPA in bouts as obese women. Similar relative differences between intensity-specific PA stratified by BMI have been reported by others [24], [25], [28]. The larger relative difference in intensity-specific PA between BMI categories than in overall PA may be explained partly by thermodynamics. Because of the greater body mass, resting energy expenditure is higher in obese compared to normal weight individuals; the greater body mass is associated with a higher metabolic cost of PA for heavier individuals. An accelerometer calibration study showed that the true MVPA intensity threshold is substantially lower for obese compared with normal weight individuals [29]. Although the metabolic cost of exercise increases with body mass, we are confident that the differences in PA between BMI categories are real and are important to public health, although care must be taken when interpreting the results for intensity-specific PA. It should also be recognized that BMI category related differences in PA might be underestimated in the present study. A study of PA using pedometers showed that a larger percentage of obese individuals increased their PA compared to those who decreased their behaviour, when monitored over 1 year [30]. If a collective behaviour of increased PA among overweight and obese in order to affect weight is picked up in the present study, this might moderate the gradient in the relationship between PA and weight status.

According to the recommendation for PA and public health set forward by the Nordic Councils of Ministers, those who are physically inactive may achieve the greatest health gains of increasing their regular PA, independent of age [31]. Although cross-sectional, the linear reduction in relative odds for being overweight or obese observed with higher levels of physical activity indicates the importance of PA to weight management. The odds of being overweight or obese differed by 53–71% between the least and most active quintile of PA and the relationship between PA and risk reductions associated with higher quintiles of PA appears to be about linear.

To our knowledge, BMI related differences in hourly activity patterns of overall PA (counts per minute) across weekdays and weekends have not been examined in large and randomly selected samples of adults and older people. A study of 108 participants by Cooper et al. (2000) showed that although the obese participants were consistently less active than non-obese participants, no significant differences were observed while participants were at work [32]. Although time at work could not be identified in the present study, the patterns of overall PA suggest that differences were least pronounced between the hours of 09∶00 and 16∶00 on weekdays and largest around midday and early afternoon on weekends. Further, compared to normal weight participants, obese participants displayed 19% lower overall PA on weekdays and a 25% lower overall PA on weekends. As the majority of the analysed sample reports working either full time (59%) or part time (11%), the observed larger relative difference in overall PA between obese and normal weight participants on weekends compared to weekdays implies that overweight and obese participants are more likely to pursuit sedentary behaviours when not constrained by work.

The findings of this study must be interpreted in light of the following limitations. We acknowledge the limitations of a cross-sectional design in establishing a causal relationship between level of activity and weight status. However, it clearly shows quantitative differences in amount of PA performed as well as differences in patterns of activity. Further, although BMI is the most commonly used measure to identify and grade overweight and obesity in populations, the method’s reliability had been questioned in individuals at the extremes of age, muscle mass, and height [33], [34]. BMI accurately predicts obesity-related morbidity and mortality in epidemiological studies [35], and it provides a reliable and robust estimate of height-independent body fatness. Another limitation is that height and weight were self-reported, which might introduce bias because of the suspected underestimating that occurs when participants self-report body weight [36]. In order to control this source of error, trained test personnel measured the weight and height of a randomly selected sub sample of the initial participants (n = 904), in a laboratory. The largest discrepancy between the self-reported and objectively measured anthropometrical data was observed for overweight women who on average underestimated their weight by 1.4 kg, indicating that a bias as a result of self-reported weight is not a threat to the validity of the present study. Among men, a small, but significant, underestimation of weight was only observed in the normal weight category (0.44 kg).

We acknowledge that accelerometers are unable to register water activities such as swimming and to accurately assess movement associated with non-ambulatory activity such as cycling [37]. To try to account for this potential source of error, participants reported the frequency and duration of cycling and swimming performed during the week of assessment. No significant differences in the total time spent performing such activities were observed between the participants in the different weight categories (data not shown) indicating that the omission of these activities from the accelerometer counts did not affect the results.

Another limitation of the present study is the relatively low participation rate. Given the declining response rates in Norway, and in other countries [38], [39], and the risk for selection bias, it is important to describe the non-responders in studies that attempt to examine samples that are representative of the general population [40], [41]; however, such analysis is rarely available [38]. Analysis of the non-responders in our study by the use of registry linkage showed that they were more likely to be either at the younger or older end of the age spectrum, unmarried and not of Norwegian origin and had lower educational and income levels, compared to the responders [42]. This has also been observed in most population-based surveys [38], [43]. Further, the sample included participants from throughout Norway, and the prevalence of overweight or obesity and other non-communicable diseases such as type 2 diabetes was similar to other national estimates. This indicates that the results from the present study have a general validity corresponding to similar studies and that the study sample was fairly representative of the general population in Norway. The study is the first epidemiological study to objectively show differences in activity patterns across weight categories and to demonstrate the contribution of PA to the prevalence of overweight and obesity in Norway.

The worldwide obesity epidemic shows no signs of abating, and, given the health risks and costs of the condition, it is crucial to understand as much as possible about the relationship between PA and weight status. Although we acknowledge that multiple factors other than PA, such as the energy intake, consummation of specific foods and beverages, alcohol use, and television watching. [44], play vital roles in the development of overweight or obesity, we believe that the findings of the present study provides additional information on the relationship between PA and BMI and suggests that there might be a particular scope for targeting the weekend as a source of increased PA among overweight and obese individuals.

### Conclusions

Both indicators of overall PA and intensity-specific PA differ between BMI categories and the risk of being overweight or obese increased with decreasing PA level. The BMI category related difference in overall PA is largest on weekends, with obese participants displaying an overall PA level 25% lower than the normal-weight participants. These findings indicate the need for planned interventions to increase the overall level of PA in the population to counteract the environmental forces that are producing a gradual weight gain in the population. The continuing use of accelerometers to monitor longitudinally the level of activity in the general population is vital for identifying the dose response relationship between PA and the prevention and treatment of overweight and obesity.
